# Joint Modeling of Response Accuracy and Time in Between-Item Multidimensional Tests Based on Bi-Factor Model

**DOI:** 10.3389/fpsyg.2022.763959

**Published:** 2022-04-11

**Authors:** Xiaojun Guo, Yuyue Jiao, ZhengZheng Huang, TieChuan Liu

**Affiliations:** ^1^School of Education Science, Gannan Normal University, Ganzhou, China; ^2^School of Humanities, Hubei University of Chinese Medicine, Wuhan, China

**Keywords:** response time, response accuracy, bi-factor model, hierarchical model, between-item multidimensional

## Abstract

With the popularity of computer-based testing (CBT), it is easier to collect item response times (RTs) in psychological and educational assessments. RTs can provide an important source of information for respondents and tests. To make full use of RTs, the researchers have invested substantial effort in developing statistical models of RTs. Most of the proposed models posit a unidimensional latent speed to account for RTs in tests. In psychological and educational tests, many tests are multidimensional, either deliberately or inadvertently. There may be general effects in between-item multidimensional tests. However, currently there exists no RT model that considers the general effects to analyze between-item multidimensional test RT data. Also, there is no joint hierarchical model that integrates RT and response accuracy (RA) for evaluating the general effects of between-item multidimensional tests. Therefore, a bi-factor joint hierarchical model using between-item multidimensional test is proposed in this study. The simulation indicated that the Hamiltonian Monte Carlo (HMC) algorithm works well in parameter recovery. Meanwhile, the information criteria showed that the bi-factor hierarchical model (BFHM) is the best fit model. This means that it is necessary to take into consideration the general effects (general latent trait) and the multidimensionality of the RT in between-item multidimensional tests.

## Introduction

With the development of modern science and technology, more and more tests are switching to computerized adaptive tests (CATs) or computer-based testing (CBT). Consequently, computers are widely used to conveniently collect item response times (RTs) in psychological and educational assessments (Shao, 2016). RTs can be an important source of information to respondents and testing. More specifically, RTs can help assess the speed of respondents, detect cheating behaviors, design better tests, and improve the accuracy of parameters estimation (van der Linden and Xiong, 2013; Fox and Marianti, 2017; Bolsinova and Tijmstra, 2018).

To make full use of RTs, much of the literature has joined RT and item response accuracy (RA) in a unidimensional item response theory (IRT) modeling framework (e.g., Meng et al., 2015; Bolsinova and Tijmstra, 2018; Guo et al., 2020). Among these, the most popular is a two-level hierarchical framework for RA and RT (van der Linden, 2007). In the two-level hierarchical framework, the first level consists of an RT model (i.e., lognormal RT model, van der Linden, 2006) and an IRT model (e.g., two-parameter logistic model). In addition, the relationships between the RT model and IRT model parameters are the second level. Compared with other popular modeling methods, Suh (2010) demonstrated that the hierarchical framework model can produce more reasonable results in terms of empirical and simulated data. These models are based on this assumption, which posits only a latent dimension to account for RT or RA (van der Linden, 2006; Klein Entink et al., 2009; Ranger and Ortner, 2011), respectively. Specifically, the dimension affecting RT is latent speed and the dimension affecting RA is latent ability. Therefore, these RT and RA models in the two-level hierarchical framework are based on a unidimensional IRT model.

In psychological and educational tests, many tests are multidimensional, either deliberately or inadvertently (Reise et al., 2010). It is inappropriate to analyze these tests on the basis of the unidimensional IRT model (Sahin et al., 2015). Consequently, some extensions based on multidimensional perspectives on the joint hierarchical modeling approach have been put forth. Man et al. (2019) proposed a hierarchical model that integrates a compensating multidimensional IRT model and a lognormal RT model using RAs and RTs. In addition, Wang et al. (2019) integrated a Multidimensional Graduated Response (CMRM) model into the hierarchical framework model to analyze multidimensional health measurement data. In these multidimensional joint hierarchical models, these RT models are all unidimensional. Nevertheless, Zhan et al. (2020) held that each latent speed should be paired with a latent ability in multidimensional tests, a multidimensional lognormal RT model was proposed based on the unidimensional lognormal RT model (van der Linden, 2006). In the multidimensional test, items are divided into between-item and within-item multidimensionality. In addition to the specific latent traits measured by the different groups of items, there is also a general latent trait that may be measured by all items in the between-item multidimensional test. Unidimensional or multidimensional IRT or RT models cannot describe this feature of between-item multidimensional tests. A suitable model is a bi-factor model that contains both general and specific latent traits. The bi-factor model hypothesizes a general latent trait, onto which all items load, and a series of orthogonal (uncorrelated) specific latent traits that load different group items (Reise, 2012). Meanwhile, the bi-factor model is valuable and widely used in RA data from psychological and educational tests (Chen et al., 2006; Rodriguez et al., 2016; Dunn and McCray, 2020). Theoretically, the latent speeds of between-item multidimensional RT data should be paired with the latent abilities of RA data (Zhan et al., 2020). Yet, currently there exists no RT model that provides a general effects measure for between-item multidimensionality in RT data. Simultaneously, there is no hierarchical framework model that integrates RT and RA for considering the general effects of between-item multidimensional tests. This research study is aiming to fill this gap in the literature.

Inspired by the work of van der Linden (2006, 2007) and Man et al. (2019), a joint hierarchical bi-factor modeling approach for between-item multidimensional RA and RT is proposed in this study. The proposed joint hierarchical bi-factor model that joined a bi-factor RT model and a bi-factor IRT model is an extension of the hierarchical modeling framework. In the bi-factor joint hierarchical modeling framework, a bi-factor RT model and a bi-factor IRT model are the first level, and the relationships between the bi-factor RT model and bi-factor IRT model parameters are the second level.

The article is organized as follows: First, the bi-factor joint hierarchical model is described. Second, a Bayesian estimation procedure is proposed and some simulation studies are used to evaluate the recovery of the parameters. Third, different hierarchical models are compared using an empirical example based on the information criteria. Finally, the article concludes with a discussion.

## Bi-Factor Hierarchical Model

In psychological and educational tests, between-item multidimensionality is found when each item measures only one latent trait. Moreover, different grouped items measured different specific latent traits, and a general latent trait was measured by all items. The nature of such tests is well described in the bi-factor model. Therefore, this study will build a joint hierarchical model of RT and RA based on the bi-factor model in between-item multidimensional tests.

### Level 1: Bi-Factor Item Response Theory Model

At the first level of the bi-factor joint hierarchical modeling framework for RA, a bi-factor IRT model (Cai et al., 2011) is specified. In the bi-factor IRT model, the probability of correctly answering an item is influenced by a weighted linear combination of general ability and several specific abilities, which is formulated as


(1) $$P\left(u_{i\,j}=1|\theta_{g\,i},\theta_{s\,i}\right)=\frac{1}{1+e\,x\,p\,\left(-\left(d_{j}+a_{g\,j}\,\theta_{g\,i}+a_{s\,j}\,\theta_{s\,i}\right)\right)}$$

where P(u_*ij*_ = 1|_*gi,si*_) is the probability of a correct answer to item *j*, *j* = 1,…,m, by person *i*, *i* = 1,…,N. *a*_*gj*_ is the discrimination of general ability for item *j*, *a*_*sj*_ is the discrimination of the *s*th specific ability for item *j*, d_j_ is the location parameter for item *j.* θ_*gi*_ and θ_*si*_ are the general ability and specific group ability for person *i*.

### Level 1: Bi-Factor Response Time Model

The lognormal RT model (van der Linden, 2006) is the most popular model for RTs. Additionally, the lognormal RT model assumes that the log-transformed RTs follow a normal distribution and are unidimensional. The latent trait speed of between-item multidimensional RT data should be paired with the latent trait ability of RA data. The multidimensional RT model proposed by Zhan et al. (2020) does not measure the general effect of all items. Therefore, a bi-factor lognormal RT model was proposed. The bi-factor lognormal RT model is


(2) $$I\,n\,T_{i\,j}=\beta_{j}-\left(\alpha_{g\,j}\,\tau_{g\,i}+\alpha_{s\,j}\,\tau_{s\,i}\right)+\xi_{i\,j},\xi_{i\,j}∼N\,\left(0,\sigma_{j}^{2}\right)$$

where τ_*gi*_ and τ_*si*_ are the general speed and specific group speed for person *i*. The item parameter _j_ denotes time-intensity for item *j*. The item parameters α_*gj*_ and α_*sj*_ are the slope parameters of the general speed τ_*gi*_ and specific speed τ_*si*_, respectively. Within Equation (2), lnT_*ij*_ is the RT of person *i* on item *j* after a log transformation. ξ_*ij*_ is the time residual for person *i* on item *j* and follow a normal distribution with variance $\sigma_{\text{j}}^{2}$ and mean 0. Moreover, the reciprocal of the variance $1/\sigma_{\text{j}}^{2}$ can also be interpreted as the time discrimination parameter for item *j*.

### Level 2: Modeling Person and Item Parameters

In the bi-factor model, there is a general assumption that the general trait and several specific traits are not correlated with each other (Cai et al., 2011; Reise, 2012). Based on the bi-factor model of RA and RT, the second level consists of the general latent trait distribution, the specific latent trait distribution, and the item parameter distribution.

The general latent trait distribution is the relationship between general ability θ_*g*i_ and speed τ_*gi*_ for the population of test-takers, which is assumed to draw from a bivariate normal distribution with mean vector μ_*I*_*g*__ and covariance matrix Σ_*P*_*g*__, such that


(3) $$\mu_{I_{g}}=\left[\mu_{\theta_{g}}\,\mu_{\tau_{g}}\right]\;\text{and}\;\Sigma_{P_{g}}=\left[\begin{array}{cc} \sigma_{\theta_{g}}^{2}\,\sigma_{\theta_{g}\,\tau_{g}} & \\ \sigma_{\theta_{g}\,\tau_{g}}\,\sigma_{\tau_{g}}^{2} & \end{array}\right]$$

In addition, the group latent trait distribution is the distribution of specific ability θ_*si*_ and speed τ_*si*_ that is also assumed to follow a multinormal distribution. The mean vector μ_*I*_*s*__ and covariance matrix Σ_*P*_*s*__ of the multivariate normal distribution are


(4) $$\mu_{I_{s}}=\left[\mu_{\theta_{s}}\,\mu_{\tau_{s}}\right]\;\text{and}\;\Sigma_{P_{s}}=\left[\begin{array}{cc} \sigma_{\theta_{s}}^{2}\,\sigma_{\theta_{s}\,\tau_{s}} & \\ \sigma_{\theta_{s}\,\tau_{s}}\,\sigma_{\tau_{s}}^{2} & \end{array}\right]$$

Finally, the dependence of item parameters is defined as a bivariate normal distribution in the second-level model. The mean vector μ_*J*_ and covariance matrix Σ_J_ are, respectively, defined as:


(5) $$\mu_{J}=\left[\mu_{d}\,\mu_{\beta }\right]\;\text{and}\;\Sigma_{J}=\left[\begin{array}{cc} \sigma_{d}^{2}\,\sigma_{d\,\beta } & \\ \sigma_{d\,\beta }\,\sigma_{\beta }^{2} & \end{array}\right]$$

Within Equations (3–5), the parameters σ_θ_*g*_τ_*g*__, σ_θ_*s*_τ_*s*__, and σ_*d*β_ represent the covariance between general ability and speeds, different specific abilities and speeds, and time-intensity β and location parameter *d*, respectively. In the person parameters, all of them mean that a positive value indicates that participants who respond to an item quicker also have a higher latent ability (van der Linden, 2007; Bolsinova et al., 2017). For the item parameter σ_*d*β_, a negative value generally reflects that the harder the item, the more time it takes.

**FIGURE 1:**
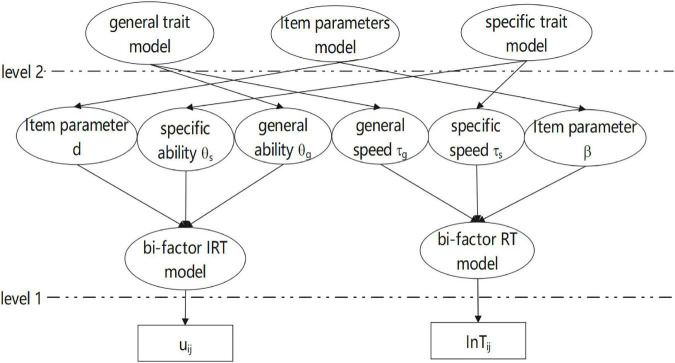
A bi-factor hierarchical model in between-item multidimensional test.

The bi-factor hierarchical model (BFHM) has been extended based on the hierarchical model proposed by van der Linden (2007) and Man et al. (2019). Figure 1 displays the graphical representation of the BFHM.

The BFHM can be simplified to a series of other hierarchical models. When the BFHM does not have general ability and speed (*a*_*gj*_ = 0 and α_*gj*_ = 0), the BFHM can be simplified to a complete multidimensional hierarchical model (CMHM) with a multidimensional RT model and a multidimensional IRT model. In CMHM, the multidimensional RT model is reduced to a unidimensional lognormal RT model (The number of dimensions of speed is fixed at 1, *s* = 1) (van der Linden, 2006) and the CMHM is transformed into a partial multidimensional hierarchical model (PMHM, Man et al., 2019). Finally, when both RT and IRT models are unidimensional models, the BFHM becomes a unidimensional hierarchical model (van der Linden, 2007).

## Estimation and Model Selection

### Bayesian Estimation Using Hamiltonian Monte Carlo Sampling

A Hamiltonian Monte Carlo (HMC) algorithm was used for model parameter estimation. The specific HMC algorithm used by Stan software is the no-U-turn sampler (Hoffman and Gelman, 2014). Compared with the Markov chain Monte Carlo algorithm, the HMC algorithm can improve efficiency and provide faster inference (Ames and Au, 2018). In addition, the users can also interact with Stan with various computing environments, including R, Python, Mathematica, and other software. All simulation data of RA and RT were generated by R version 4.1.0. Two chains with thinning of two were executed using 40,000 total iterations. All parameter estimates and standard deviations from the posterior densities were computed using the final 20,000 iterations. Rstan package was utilized to execute the HMC algorithm for parameter estimation. The potential scale reduction factor (PSRF) was used for evaluating convergence for all model parameters and required less than 1.1. (Brooks and Gelman, 1998).

### Identifying Restrictions

To accurately identify the BFHM, the parameters should be fixed to μ_θ_*g*__ = μ_τ_*g*__ = μ_θ_*s*__ = μ_τ_*s*__ = 0, and $\sigma_{\theta_{g}}^{2}\,\left[c\,p\,s\,b\,r\,e\,a\,k\right]=\sigma_{\tau_{g}}^{2}=\sigma_{\theta_{s}}^{2}=\sigma_{\tau_{s}}^{2}=1$ (van der Linden, 2007; Cai et al., 2011).

### Prior Distributions

The prior distribution for the item parameters *a*_*gj*_, *a*_*sj*_, α_*gj*_, α_*sj*_ and 1/σ_j_ all follow the left truncated normal distribution *N*(0,1) that is conditioned or regulated to be in the interval (0, + ∞). Due to identifying restrictions, the correlation ρ_θτ_ is equal to the covariance σ_θτ_. The correlation matrices Σ_*P*_*g*__ and Σ_*P*_*s*__ have a Cholesky factorization Σ_*P*_*g*__ = Σ_*P*_*s*__ = Ω = *LL*′, where *L* is a lower triangular matrix. The prior distribution on *L* follows a Cholesky Lewandowski-Kurowicka-Joe (LKJ) correlation distribution *L*∼*lkj*_*corr*_*cholesky*(η) in Stan software. In Stan software, the Cholesky LKJ correlation distribution is defined as *LkjCholesky*(L|η)∝⌈*J*⌉det(*LL*^*T*)^η−1^^ and the parameter η is set 1. Moreover, the item parameters d_j_ and β_j_ are assumed to follow a multivariate normal distribution. The covariance matrix *Sigma*_I_ of the multivariate normal distribution can be broken down into $Sigma_{\text{I}}=\left[\begin{array}{cc} \sigma_{d} & 0\\ 0 & \sigma_{\beta } \end{array}\right]\;*\Omega *\;\left[\begin{array}{cc} \sigma_{d} & 0\\ 0 & \sigma_{\beta } \end{array}\right]$, where Ω is a correlation distribution and the lower triangular matrix *L* of Ω follows the distribution of the Cholesky LKJ correlation distribution *L*∼*lkj*_*corr*_*cholesky*(1). The hyper-priors of the mean vector distribution are μ_*d*_∼*N*(0,0.5), μ_β_∼*N*(4,0.5), while the standard deviation σ_β_ and σ_*d*_ follow left truncated normal distribution *N*(0,1) and truncate above 0.

### Model Fit for the Hierarchical Models

The widely available information criterion (WAIC, Vehtari et al., 2017) and leave-one-out cross-validation (LOO, Vehtari et al., 2017) are considered for purposes of model checking and model comparison in this study by Stan software. Luo and Al-Harbi (2017) indicated that the information criterion WAIC and LOO are better than the traditional model fitting index in the IRT model, such as the deviance information criterion (DIC), Akaike’s information criterion (AIC), and Bayesian information criterion (BIC). Meanwhile, the information criterion WAIC and LOO can be calculated by Rstan and LOO packages in R software.

## Simulation Study

### Design of the Simulation Study

To verify the parameter recovery with the proposed estimation method, the most complex model of BFHM was selected as the simulation model. In this study, the simulated data included two conditions for evaluating the parameters recovery of items and persons. In addition, the dimension of specific ability and speed is fixed to 3. The group items of each specific ability and speed are equal. In simulated conditions, two levels of the number of examinees were considered (*N* = 500, 1,000) and two-level test length was simulated (*m* = 30, 60). For item parameters *a*_*gj*_, *a*_*sj*_, α_*gj*_, α_*sj*_ and 1/σ_j_, these parameters were sampled from a left truncated normal distribution *N*(0,1) and truncated above 0. Item parameters d_j_ and β_j_ were drawn from a bivariate normal distribution. The mean vector of the bivariate normal distribution was set as 0 and 4. Moreover, the corresponding variances were, respectively, fixed to 1 and 0.25, and the covariance was set to –0.25. For the person parameters, the general ability θ_gi_ and speed τ_*gi*_, specific ability θ_si_ and speed τ_*si*_, all followed a bivariate normal distribution. The corresponding correlation coefficients were sampled from a uniform distribution U(−1,1). Finally, in the bi-factor IRT model (Equation 1), the relevant parameters were substituted into the model and the probability was calculated and compared with a random 0–1. If it was greater than or equal to the random number, the answer was correct as 1, otherwise, the answer error was 0. Moreover, the mean of the logarithmic RT was calculated by substituting the relevant parameters into Equation (2) and combined with the variance $\sigma_{\text{j}}^{2}$, the logarithmic RT (InT_ij_) was generated according to the normal distribution.

In our simulation study, there are a total of 2 × 2 = 4 crossed conditions. Each condition was replicated 30 times. The mean squared error (MSE) and the average bias (Bias) were used to evaluate the item and person parameters recovery.


(6) $$M\,S\,E\,\left(\hat{\xi }\right)=\frac{\sum_{r=1}^{R}\sum_{j=1}^{m}{\left(\hat{\xi }-\xi \right)}^{2}}{R*m}$$

(7) $$B\,i\,a\,s\,\left(\hat{\xi }\right)=\frac{\sum_{r=1}^{R}\sum_{j=1}^{m}\left(\hat{\xi }-\xi \right)}{R*m}$$

Where $\hat{\xi }$ and ξ are the estimated and true values of model parameters, respectively. *R* is the number of replications and *m* is the test length or the number of examinees.

### Results of the Simulation Study

The estimated results of the item parameters are shown in Table 1. In different item parameters, the MSE of item parameters decreased as the number of examinees *N* increased. Meanwhile, the results of the item parameters estimation of the bi-factor RT model were better than those of the bi-factor IRT model. Specifically, the MSE values of the two discrimination parameters of the bi-factor IRT model were in the range of approximately 0.040 to close to 0.010 with the number of examinees from 500 to 1,000. Under the same conditions, the MSE of the location parameter was decreased from 0.111 to 0.01. For the bi-factor RT model, the MSE of all item parameters was less than 0.007 in all conditions. Moreover, the absolute Bias of all item parameters was below 0.04.

**TABLE 1:** MSE and Bias for the item and person parameters.

Model parameters		*N* = 500, *m* = 30		*N* = 1,000, *m* = 30		*N* = 500, *m* = 60		*N* = 1,000, *m* = 60	
		MSE	Bias	MSE	Bias	MSE	Bias	MSE	Bias
Item parameters	*a_g_*	0.040	0.013	0.017	0.017	0.033	0.033	0.017	0.017
	*a_s_*	0.052	0.040	0.026	0.020	0.030	0.016	0.011	0.008
	d	0.111	0.021	0.010	0.033	0.019	–0.036	0.010	–0.021
	α_*g*_	0.007	0.036	0.003	0.014	0.006	0.024	0.002	–0.002
	α_*s*_	0.005	0.012	0.003	0.020	0.003	0.010	0.002	0.000
	σ	0.002	0.002	0.001	0.002	0.002	–0.001	0.001	–0.002
	β	0.008	0.018	0.003	–0.011	0.005	–0.009	0.002	–0.007
Person parameters	θ_*g*_	0.323	–0.006	0.268	–0.008	0.200	0.004	0.213	0.044
	θ_*s* = 1_	0.464	0.014	0.458	–0.001	0.312	0.039	0.323	–0.021
	θ_*s* = 2_	0.490	0.015	0.497	–0.005	0.343	0.059	0.309	–0.030
	θ_*s* = 3_	0.525	–0.008	0.530	–0.011	0.338	0.008	0.300	–0.018
	τ_*g*_	0.081	0.021	0.075	–0.002	0.046	–0.019	0.046	–0.008
	τ_*s* = 1_	0.105	–0.003	0.103	0.010	0.067	0.003	0.068	–0.012
	τ_*s* = 2_	0.119	–0.019	0.152	–0.003	0.071	0.016	0.063	0.024
	τ_*s* = 3_	0.152	0.017	0.120	–0.017	0.062	0.012	0.058	0.005

Alternatively, Table 1 also shows the results of the person parameters. The MSE of person parameters decreased as the test length *m* increased and the person parameters of the bi-factor RT model were better than that of the bi-factor IRT model. In different ability parameters, the general ability decreased from 0.323 to 0.213, and the different specific abilities were reduced from approximately 0.5 to near 0.3 with increasing test length. The corresponding different speed parameters were reduced from about 0.1 to about 0.06. Meanwhile, the absolute Bias of the person parameters fluctuated around 0.01.

Overall, the obtained results indicate that the HMC algorithm can effectively estimate all parameters.

## Empirical Example

### Data Set Description

Data from the partial items of the Raven’s Standard Progressive Matrices (SPM) were used to fit the BFHM, CMHM, and PMHM. The SPM includes five subtests (A–E) and 12 items in each subtest. This study collected 10 items in each of the subtests A, C, and D through E-prime 2.0, and the time limit for answering was 30 min. Items of the 3 subtests were presented in random order. Meanwhile, the participants cannot skip the item before answering and cannot be allowed to be returned. The three-dimensional slope parameter-loading pattern is displayed as


(8) $$S=\left[\begin{array}{c} 1,1,1,1,1,1,1,1,1,1,0,0,0,0,0,0,0,\\ 0,0,0,0,0,0,0,0,0,0,0,0,0\\ 0,0,0,0,0,0,0,0,0,0,1,1,1,1,1,1,1,\\ 1,1,1,0,0,0,0,0,0,0,0,0,0\\ 0,0,0,0,0,0,0,0,0,0,0,0,0,0,0,0,0,\\ 0,0,0,1,1,1,1,1,1,1,1,1,1 \end{array}\right]$$

## Results

The results of the information criteria under the different hierarchical models are presented in Table 2. According to the values of WAIC and LOO, results showed that the value of the BFHM was the smallest, followed by the CMHM, and finally the PMHM (Man et al., 2019). Therefore, the BFHM is the best model to fit the empirical data. In other words, general effects (general latent trait) and the multidimensionality of RTs meet the need of the between-item multidimensional test.

**TABLE 2:** The information criteria under the different hierarchical models.

Information criteria	Model	RA	RT	Total
WAIC	BFHM	5,037.8	12,560.2	**17,598**
	CMHM	5,043.5	12,740.5	17,784
	PMHM	5,078.2	13,167.8	18,246
LOO	BFHM	5,052.6	12,598.4	**17,651**
	CMHM	5,059.0	12,754.9	17,813.9
	PMHM	5,061.7	13,171.6	18,233.3

*BFHM, the bi-factor hierarchical models; CMHM, the complete multidimensional hierarchical model; PMHM, the partial multidimensional hierarchical model. RA, response accuracy; RT, response time.*

Finally, the structural parameters of the item and person parameters in the BFHM were as follows: The mean of item parameters d_j_ and β_j_ were μ_*d*_ = 3.132 and μ_β_ = 2.460. The item covariance matrix parameters were $\sigma_{d}^{2}=2.743$, $\sigma_{\beta }^{2}=0.449$, and $\sigma_{d\,\beta }^{2}=-0.922$, 95%*CI* = [−1.620,−0.498]. The covariance conversion to correlation coefficient is ρ_*d*β_ = -0.831. This means that the more difficult the item, the more time it takes. In addition, the correlation coefficient of each specific ability and speed was close to 0 and the confidence interval included 0. That is, each specific ability and speed was independent of the other. However, the correlation coefficient was ρ_θ_*g*_τ_*g*__ = −0.181 between general ability and speed, and the confidence interval was 95%CI=[−0.370,−0.011]. The negative correlation between ability and speed has also been reported in other studies (e.g., van der Linden and Fox, 2015; Fox and Marianti, 2016). This result may be related to the test being non-high-stakes and lacking motivation.

## Discussion

With the popularity of CBT, it is easier to collect item RTs in psychological and educational assessments. RTs can provide an important source of information to respondents and tests. To make full use of RTs, researchers have devoted a lot of effort to developing an appropriate RT statistical model. Most of the proposed models posit a unidimensional latent speed to account for RTs in tests. In psychological and educational tests, many tests are multidimensional, either deliberately or inadvertently. It is not appropriate to analyze these tests based on the unidimensional joint hierarchical modeling approach. Zhan et al. (2020) proposed a multidimensional lognormal RT model, but they are not modeled jointly with RA. In addition, Man et al. (2019) proposed a joint-modeling approach that integrates compensatory multidimensional IRT model and unidimensional lognormal RT model. The joint-modeling approach can only be considered as a PMHM. However, in addition to the specific latent traits measured by the different groups of items, there is also a general latent trait that may be measured by all items in the between-item multidimensional test. Unidimensional or multidimensional IRT and RT models cannot describe this feature. Simultaneously, there is no hierarchical framework model that integrates RTs and RAs into the joint model framework and takes into account the general effects of between-item multidimensional tests. Therefore, a bi-factor joint hierarchical modeling approach for between-item multidimensional RAs and RTs is proposed in this study. Meanwhile, the parameters of the bi-factor joint hierarchical model can be performed well using the HMC algorithm in simulation. Based on the two fitting indexes of WAIC and LOO, the application of empirical data showed that the BFHM is the best fit model. This means that it is necessary to consider the general effects (general latent trait) and the multidimensionality of RTs in between-item multidimensional tests.

Some other issues should also be further considered. First, the high-order model (Huang et al., 2013) and testlet model (Zhan et al., 2018) both also consider the general effect. Under certain conditions, the two models are equivalent to the bi-factor model. It is necessary to compare the fit of the three models in RA and RT based on joint hierarchical modeling in the follow-up study. Second, the bi-factor RT model is based on the lognormal RT model (van der Linden, 2006). However, item RTs do not always follow a lognormal distribution. Therefore, some other distribution models should be considered, such as Shifted Wald distribution (Anders et al., 2016) and the semi-parameter model (Wang et al., 2013). Finally, the joint hierarchical model cannot fully explain the relationship between the RT and accuracy (e.g., Meng et al., 2015; Guo et al., 2020). Therefore, a dependent joint hierarchical model can be obtained with some extensions.

## Data Availability Statement

The original contributions presented in the study are included in the article/Supplementary Material, further inquiries can be directed to the corresponding author/s.

## Author Contributions

XG: design of the study, data analysis, manuscript writing, and revision. YJ: manuscript writing, and revision. ZH: preliminary idea construction, manuscript revision, and proofreading. TL: manuscript revision, and proofreading. All authors contributed to the article and approved the submitted version.

## Conflict of Interest

The authors declare that the research was conducted in the absence of any commercial or financial relationships that could be construed as a potential conflict of interest.

## Publisher’s Note

All claims expressed in this article are solely those of the authors and do not necessarily represent those of their affiliated organizations, or those of the publisher, the editors and the reviewers. Any product that may be evaluated in this article, or claim that may be made by its manufacturer, is not guaranteed or endorsed by the publisher.
