# Supplementary material for: Joint Modeling of Response Accuracy and Time in Between-Item Multidimensional Tests Based on Bi-Factor Model
Source: Front Psychol. 2022 Apr 11;13:763959. doi: 10.3389/fpsyg.2022.763959 (PMC9035624; doi:10.3389/fpsyg.2022.763959)
Supplement: Supplementary file 1 [file Table_1.DOCX]

Supplementary Material(stan syntax)

// The bi-factor hierarchical model

data {

int<lower=1> m;

int<lower=1> N;

int<lower=1> tt;

int<lower=1,upper=m> jj[tt];

int<lower=1,upper=N> ii[tt];

int<lower=0,upper=1> RA[tt];

vector[tt] RT;

int<lower=1> D;

int<lower=1,upper=D>Fs[tt];

}

parameters {

vector<lower=0>[m] a1;

vector<lower=0>[m] a2;

vector<lower=0>[m] alpha1;

vector<lower=0>[m] alpha2;

vector<lower=0>[m] siga;

vector<lower=0> [2] vbb;

vector[2] bbeta[m];

matrix[2, N] z_th1;

matrix[2, N] z_th2;

matrix[2, N] z_th3;

matrix[2, N] z_th4;

vector[2] mubb;

cholesky_factor_corr[2] L_th1;

cholesky_factor_corr[2] L_th2;

cholesky_factor_corr[2] L_th3;

cholesky_factor_corr[2] L_th4;

cholesky_factor_corr[2] L_bb;

}

transformed parameters{

vector[tt] eta;

vector[tt] mutt;

vector[tt] sgtt;

matrix[N,D] th;

matrix[N,D] tao;

corr_matrix[2] corth1= L_th1 * L_th1';

corr_matrix[2] corth2= L_th2 * L_th2';

corr_matrix[2] corth3= L_th3 * L_th3';

corr_matrix[2] corth4= L_th4 * L_th4';

corr_matrix[2] corbb= L_bb * L_bb';

matrix[N,2] thtao1;

matrix[N,2] thtao2;

matrix[N,2] thtao3;

matrix[N,2] thtao4;

vector[2] vth;

vth[1]=1;

vth[2]=1;

thtao1 = (diag_pre_multiply(vth, L_th1) * z_th1)' ;

thtao2 = (diag_pre_multiply(vth, L_th2) * z_th2)' ;

thtao3 = (diag_pre_multiply(vth, L_th3) * z_th3)' ;

thtao4 = (diag_pre_multiply(vth, L_th4) * z_th4)' ;

th[1:N,1]=thtao1[1:N,1];

th[1:N,2]=thtao2[1:N,1];

th[1:N,3]=thtao3[1:N,1];

tao[1:N,1]=thtao1[1:N,2];

tao[1:N,2]=thtao2[1:N,2];

tao[1:N,3]=thtao3[1:N,2];

for(i in 1:tt){

eta[i]=a1[jj[i]]*th[ii[i],Fs[i]]+a2[jj[i]]*thtao4[ii[i],1]+bbeta[jj[i],1];

sgtt[i]=1/siga[jj[i]];

mutt[i]=bbeta[jj[i],2]-(alpha1[jj[i]]*tao[ii[i],Fs[i]]+alpha2[jj[i]]*thtao4[ii[i],2]);

}

}

model {

a1 ~ normal(0,1);

a2 ~ normal(0,1);

alpha1 ~ normal(0,1);

alpha2 ~ normal(0,1);

L_th1~lkj_corr_cholesky(2);

L_th2~lkj_corr_cholesky(2);

L_th3~lkj_corr_cholesky(2);

L_th4~lkj_corr_cholesky(2);

L_bb~lkj_corr_cholesky(2);

to_vector(z_th1)~normal(0,1);

to_vector(z_th2)~normal(0,1);

to_vector(z_th3)~normal(0,1);

to_vector(z_th4)~normal(0,1);

mubb~normal(0,1);

target+=normal_lpdf(vbb|0,1)-normal_lpdf(0|0,1);

target+=normal_lpdf(siga|0,1)-normal_lpdf(0|0,1);

bbeta ~ multi_normal_cholesky(mubb, diag_pre_multiply(vbb, L_bb));

RT~normal(mutt,sgtt);

RA~bernoulli_logit(eta);

}

generated quantities{

vector[tt] log_lik;

for(i in 1:tt){

real py;

py=inv_logit(eta[i]);

log_lik[i]=bernoulli_lpmf(RA[i]|py)+ normal_lpdf(RT[i]|mutt[i],sgtt[i]);

}

}
